# Surgical Workshop on Liver Surgery Using Isolated Perfused Livers in Moulded Casts of the Upper Abdomen

**DOI:** 10.1155/1995/93925

**Published:** 1995

**Authors:** Gadžijev Eldar, Ravnik Dean, Sojar Valentin

**Affiliations:** 1 University Medical Centre Ljubljana Slovenia; 2 Institute for Anatomy Medical Faculty University of Ljubljana Slovenia

## Abstract

The basic training in liver surgery on isolated perfused livers used at the workshop in the First Surgical Course of the Alps—Adriatic Hepatobiliary School is presented. The methods for the excision, preservation, perfusion and preparation of the liver are described, as is the manner of manufacturing the upper abdomen moulded casts, into which an isolated perfused liver is placed for training. The methods proved to be sufficiently successful, enabling participants to perform basic liver surgery like an intraoperative ultrasound investigation, as well as liver dissection techniques, liver suturing, segmental resection and even hepatectomy. Some technical improvements are proposed for future surgical workshops, such as washing out the blood from the liver, and a triple perfusion.


*HPB Surgery*, 1995, Vol. 9, pp. 19–24
Reprints available directly from the publisher
Photocopying permitted by license only

© 1995 OPA (Overseas Publishers Association)
Amsterdam B.V. Published in The Netherlands
by Harwood Academic Publishers GmbH
Printed in Malaysia

### Surgical Workshop on Liver Surgery Using Isolated Perfused Livers in Moulded Casts of the Upper Abdomen

GADŽIJEV ELDAR*, RAVNIK DEAN and SOJAR VALENTIN*

*University Medical Centre Ljubljana, Slovenia, Institute for Anatomy, Medical Faculty, University of Ljubljana

(*Received November 28, 1994*)

The basic training in liver surgery on isolated perfused livers used at the workshop in the First Surgical Course of the Alps—Adriatic Hepatobiliary School is presented. The methods for the excision, preservation, perfusion and preparation of the liver are described, as is the manner of manufacturing the upper abdomen moulded casts, into which an isolated perfused liver is placed for training. The methods proved to be sufficiently successful, enabling participants to perform basic liver surgery like an intraoperative ultrasound investigation, as well as liver dissection techniques, liver suturing, segmental resection and even hepatectomy. Some technical improvements are proposed for future surgical workshops, such as washing out the blood from the liver, and a triple perfusion.

KEY WORDS: Liver surgery training isolated perfused liver moulded casts of the upper abdomen

### INTRODUCTION

Surgical procedures on the liver are accompanied by specific problems, which require particular skills from surgeons. Since abdominal surgery was established, liver surgery has been perceived as a professional challenge; however, surgeons were quite afraid to perform it. Nowadays, general surgeons are still reluctant to perform liver operations because of the bleeding and difficulties in stopping it.

A good knowledge of detailed liver anatomy[1,2], the advantages of intraoperative ultrasound investigation[3,4] and appropriate professional training can help surgeons to perform liver resections safely and effectively[5].

A workshop on basic liver surgery was included in the programme of the First Surgical Course of the Alps—Adriatic Hepatobiliary School. Training on cadavers was planned, but we soon faced problems regarding an appropriate number of cadavers per day. This led to the idea of preparing isolated cadaveric livers and using them as models for such training. It was agreed with the Institute for Pathology that models for training would be prepared from cadaveric livers obtained during autopsies.

For basic training in liver surgery on isolated cadaveric livers, the following exercises were planned: anatomic orientation, ultrasound investigation, liver dissection techniques, liver suturing, necrectomy, segmental resections and hepatectomies. Other important procedures, like liver mobilisation, vascular occlusion, perihepatic packing and dissection of the structures in the hepatoduodenal ligament, were intended to be performed on cadavers.

### MATERIALS AND METHODS

### Excision of the Liver and its Conservation

During an autopsy, the liver was mobilised from the hepatic fossa; the inferior caval vein was transected at the level of the renal veins, while the hepatoduodenal ligament was cut as close to the duodenum as possible. The suprahepatic vena cava and part of the diaphragm were cut out and the liver was removed.

*Address for correspondence*: Gadzijev Eldar, Department of Gastroenterological Surgery, University Medical Centre Ljubljana, Zaloska 7, 61000 Ljubljana, Slovenia.

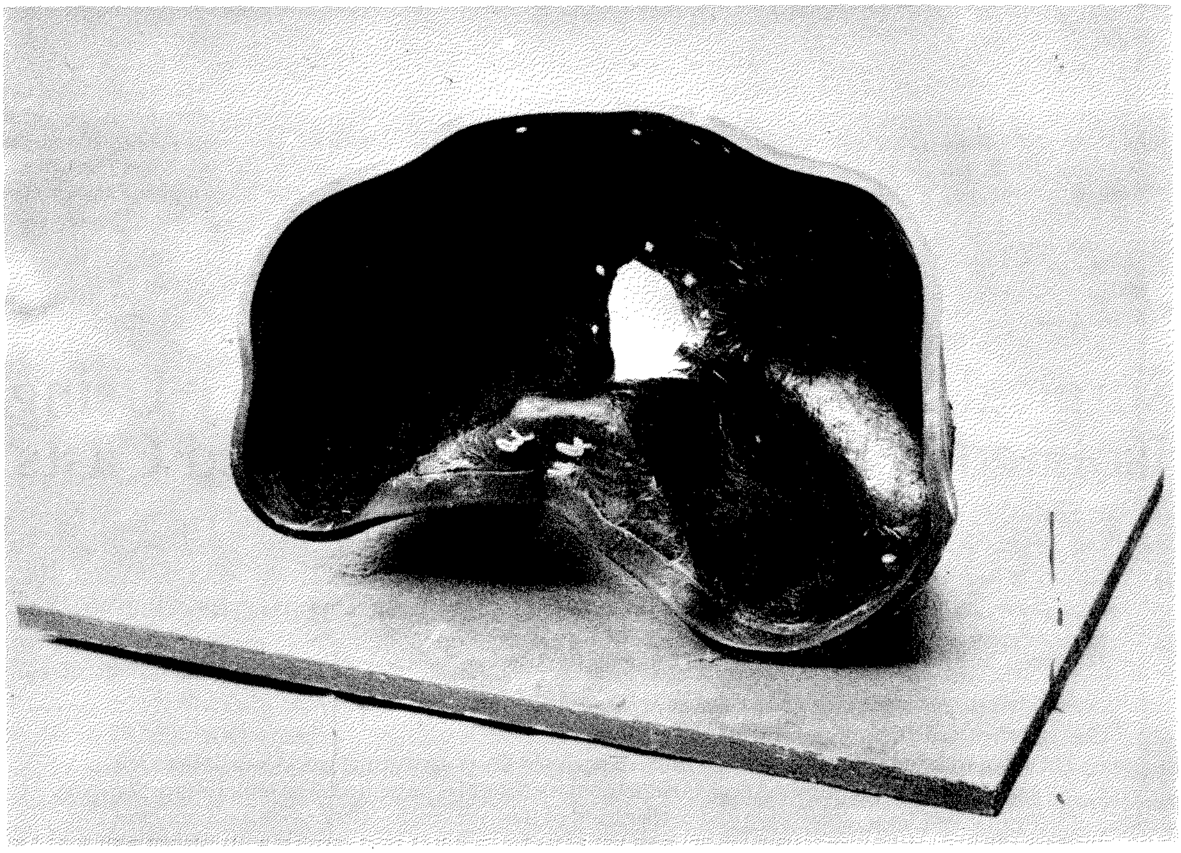

**Figure 1** A moulding cast of the upper abdomen ready for the surgical workshop.

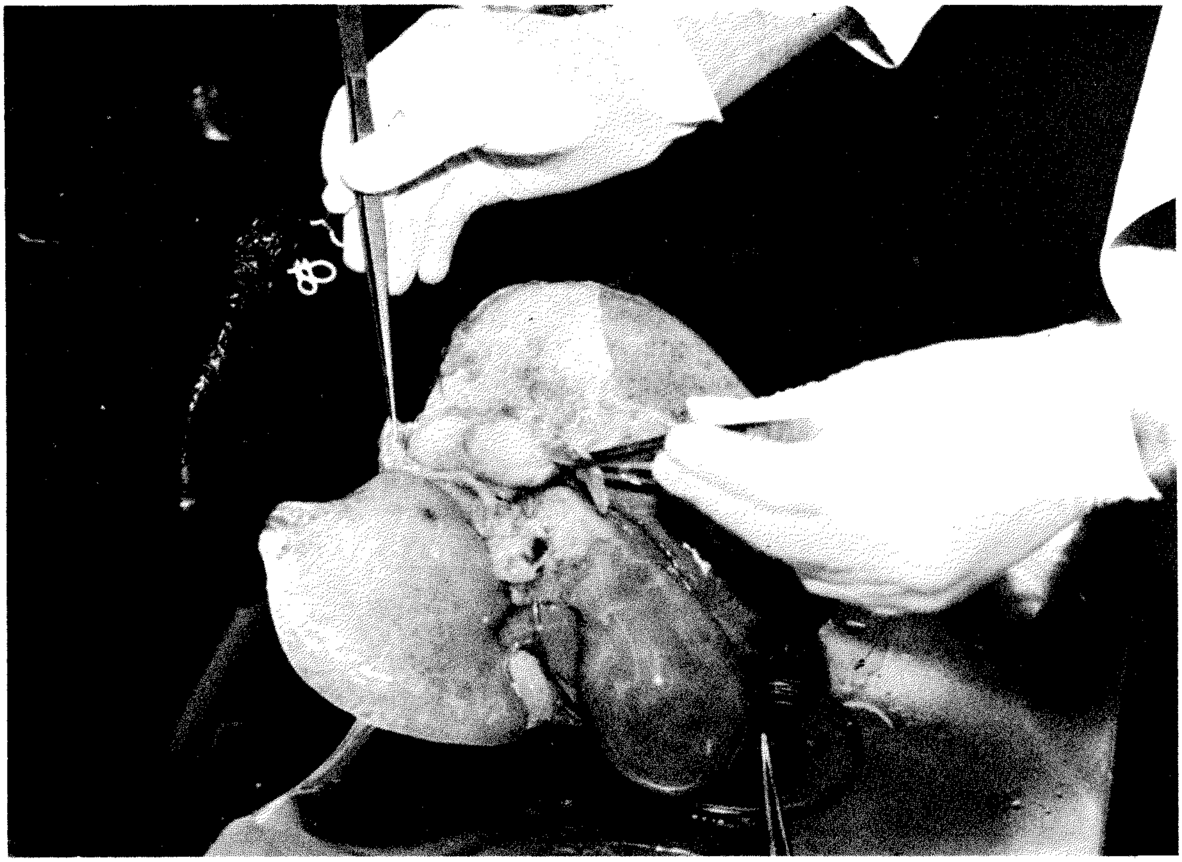

**Figure 2** An isolated liver with perfusion through a cannula inserted in the portal vein.

Blood was left for a couple of minutes to ooze out from the liver, which was then placed in a specially prepared moulding cast, and put into a freezer to be frozen at −25 degrees C.

### Preparation of Moulding Casts Simulating the Upper Abdomen

One of the cadaveric preparations of the upper abdomen, used as a preparation during anatomy lessons, served as a mould for a detailed cast, which was made by placing the polyester laminate into the interior of the preparation. Several similar casts were produced by using the exterior of the first such mould as a pattern. The models were painted with polyurethane paint and the sharp edges of the laminate were protected. The prominence of the inferior caval vein was cut, several holes were drilled and slings were mounted to enable fixation (Figure 1).

### Final Preparation of the Isolated Liver for Perfusion and Training

The liver was defrosted twelve hours before the beginning of the training programme. A modification of the bench preparation was performed: the portal vein was isolated and cannulated, the artery and the common bile duct were ligated, and the caval vein was sutured on both sides. As a rule, the right adrenal vein was ligated. After these procedures, the liver was infused with water and methylene blue through a cannula inserted in the portal vein. Additional ligatures or sutures were often needed to close the leaking points (Figure 2). Then the liver was put into the cast of the upper abdomen by placing the caval vein in the aperture of the mould. The liver, fixed to the slings and holes in the cast, became bloated when filled with water through cannulation (Figure 3). All these procedures prepared the liver for training of an intraoperative ultrasound examination and some basic surgical procedures.

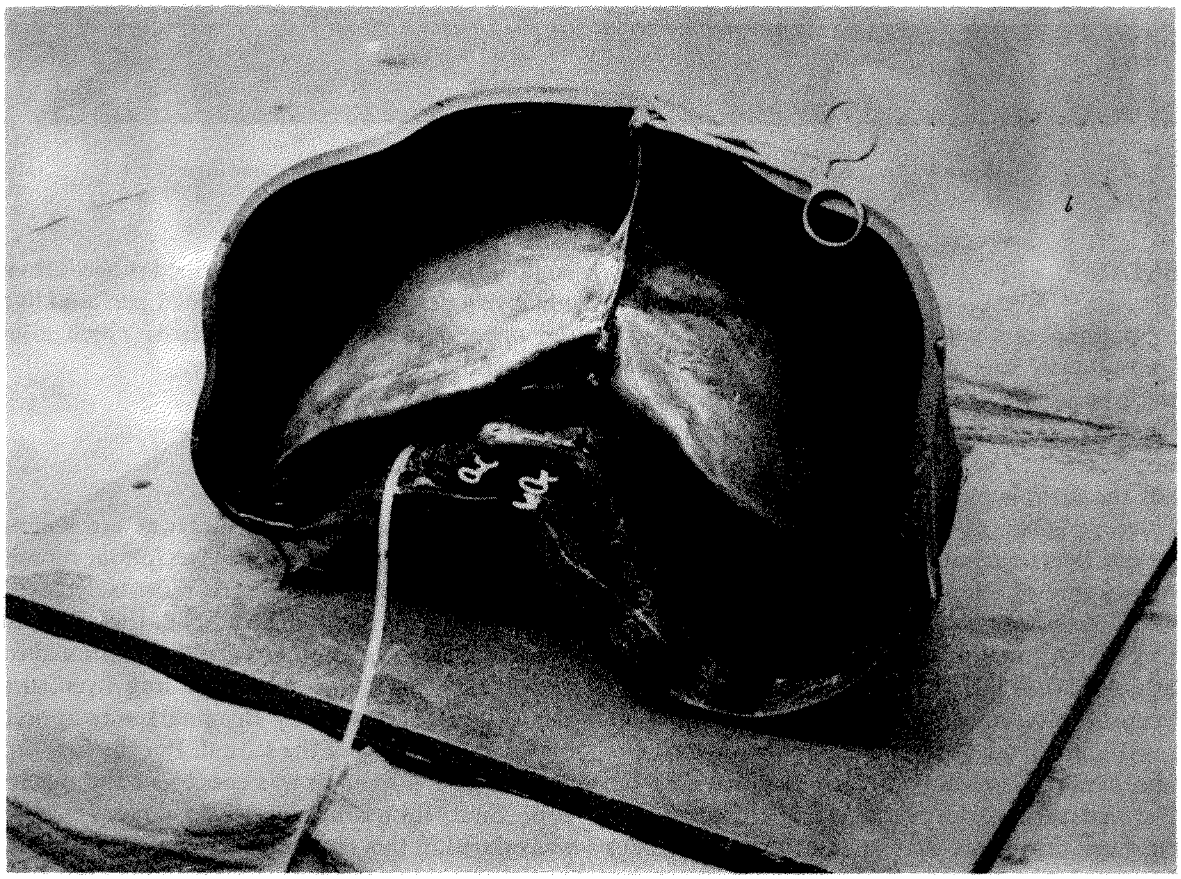

**Figure 3** The isolated perfused liver placed in the moulding cast of the upper abdomen.

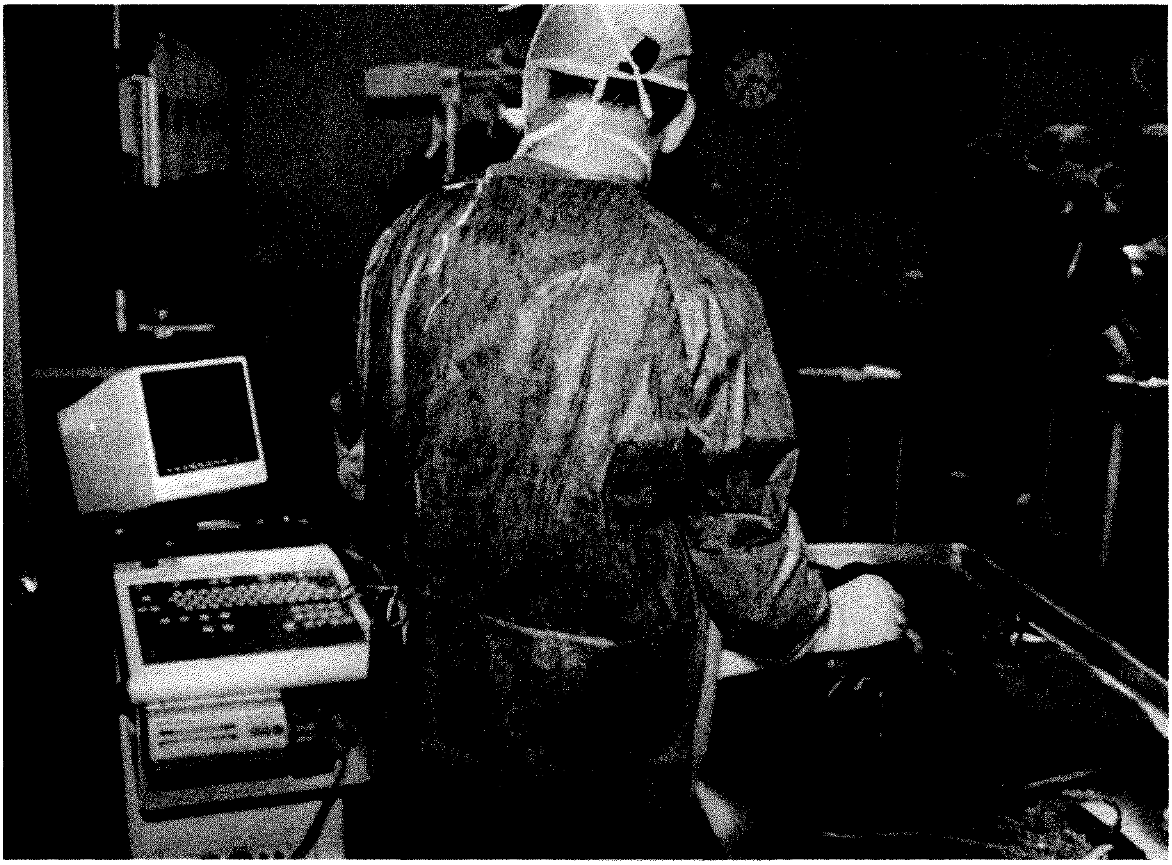

**Figure 4** Training in intraoperative ultrasound investigation on the isolated perfused liver.

### RESULTS

The moulding cast with the liver perfused through the portal vein was placed on an autopsy table. Water continually flowed out. First to be evaluated was an ultrasound investigation as practically used intraoperatively. The branches of the portal vein, as well as the main hepatic veins and the inferior caval vein, were visible. Given the recognised anatomic structures, liver segments could be determined (Figure 4). A puncture of the portal branches or the hepatic vein was attempted by using a special probe needle with a guide. A coloured fluid in the syringe proved the success of puncturing. An incision performed over the liver capsule was followed by different dissection techniques (Figure 5) applying ligatures, sutures and clips on the exposed structures. Various sorts of liver sutures were then used. After crushing a selected part of the liver tissue, necrectomy was practised using dissectors. After exposing the inner liver structures and ligating them, segmentectomy and hepatectomy could be performed. All the described procedures were effectively performed on the isolated perfused liver in the upper abdomen moulding cast, which allowed successful training in liver surgery.

### DISCUSSION

Nowadays, more and more surgeons are willing to perform liver surgery. As surgical technique represents an art form[6], proper training is most welcome. The Alps—Adriatic Hepatobiliary School was organised in Ljubljana, Slovenia to provide for additional training of as many surgeons performing liver surgery as possible. Professor Stig Bengmark was appointed honorary president of the school. For the first time, a liver surgery workshop was conceived and a suitable opportunity for training basic liver surgery techniques was offered in this region. The initial idea of training on cadavers had to be more or less abandoned because of the uncertainty and unpredictability regarding an

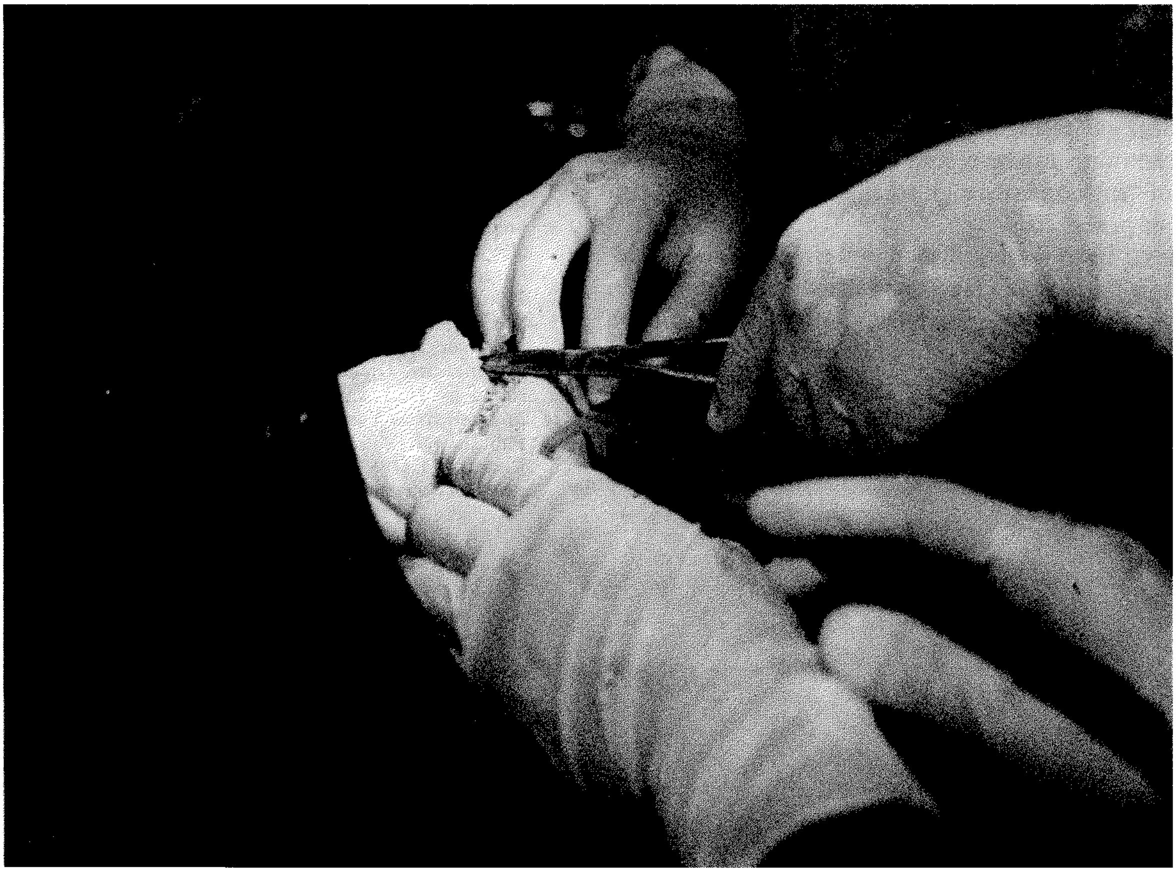

**Figure 5** Training in dissection techniques on the isolated perfused liver.

appropriate supply of cadavers for such a workshop. At least five isolated livers per day had to be arranged in order to enable sufficient practice for all participants of the surgical course. Months before the school had begun, livers were collected during routine autopsies, with the consent of the National Ethics Committee and the Institute for Pathology. Freezing the liver after excision from a cadaver and defrosting it 12 hours before training seemed to be an effective method of providing and storing material for the workshop. Proper preparation of the liver on a bench with cannulation of the portal vein and accurate ligating and suturing of the caval vein and the structures in the hepatoduodenal ligament proved to be an important step in adequately preparing the liver for training. Although ultrasound investigation as performed intraoperatively was suitable for the isolated perfused liver, air bubbles within the liver sometimes prevented clear visualization. In the future, we should entirely exsanguinate the liver with perfusion after removing it from a cadaver and before storing it in a freezer. This seemed quite important, because a good visualization of internal liver structures is essential for an accurate anatomical orientation of the liver.

Basic liver dissection techniques, the management of internal structures, liver suturing and even resections could be exercised on isolated perfused liver. Permanent perfusion through the liver model during the surgical work was important, since it gave an impression of the blood flow.

For the next workshop, cannulation of all three systems in the hepatoduodenal ligament is planned to make a more effective impression of a "lively liver". Training on the liver models could give the false impression that liver surgery is very simple—therefore we should prepare liver mould in such a way that the possibility and probability of real "bleeding" could be quite evident and should not be neglected.

### CONCLUSION

A training programme in liver surgery performed on isolated perfused liver is one of the most beneficial surgical exercises, in particular because the internal liver anatomy can be precisely recognized and a proper use of "intraoperative" ultrasound investigation can also be practiced.

Basic liver resection techniques can also be adequately performed. New models, which would even more resemble a real, live liver, should in the future be taken into consideration to enhance the impression of bleeding possibilities during liver surgery.

### REFERENCES

1. Couinaud, C. (1954) Lobes et segments hepatiques. *Presse Medicale*, **62**, 709–712.
2. Bismuth, H. (1982) Surgical anatomy and anatomical surgery of the liver. *World Journal of Surgery*, **6**, 3–9.
3. Traynor, O., Castaing, D. and Bismuth, H. (1988) Peroperative ultrasonography in surgery of hepatic tumours. *British Journal of Surgery*, **75**, 197–202.
4. Solomon, M. J., Stephen, M. S., White, G. H. and Eyers, A. A. (1992) A new classification of hepatic territories using intraoperative ultrasound. *The American Journal of Surgery*, **163**, 336–338.
5. Scheele, J. (1989) Segment-orientated resection of the liver: Rationale and technique. In Hepatobiliary and pancreatic malignancies. Diagnosis, medical and surgical management, edited by N. J. Lygidagis and G. N. Tygat, pp. 219–247. *Stuttgart*, New York: Georg Thieme Verlag, Thieme Medical Publishers.
6. Foster, J. H. (1989) Liver resection techniques. *Surgical Clinics of North America*, **69** (2), 235–249.

